# Adapting the chemical unfolding assay for high-throughput protein screening using experimental and spectroscopic corrections

**DOI:** 10.1016/j.ab.2018.08.027

**Published:** 2018-12-15

**Authors:** J. Alaina Floyd, Christine Siska, Rutilio H. Clark, Bruce A. Kerwin, Jeremy M. Shaver

**Affiliations:** Just Biotherapeutics, Inc., 401 Terry Ave N., Seattle, WA, 98109, USA

**Keywords:** Chemical unfolding, High-throughput, Antibody, Denaturation, Fluorescence, Scatter correction, Equilibration time

## Abstract

The chemical unfolding (denaturation) assay can be used to calculate the change in the Gibbs free energy of unfolding, ΔG, and inflection point of unfolding, to collectively inform on molecule stability. Here, we evaluated methods for calculating the ΔG across 23 monoclonal antibody sequence variants. These methods are based on how the measured output (intrinsic fluorescence intensity) is treated, including utilizing (a) a single wavelength, (b) a ratio of two wavelengths, (c) a ratio of a single wavelength to an area, and (d) a scatter correction plus a ratio of a single wavelength to an area. When applied to the variants, the three ratio methods showed comparable results, with a similar pooled standard deviation for the ΔG calculation, while the single-wavelength method is shown as inadequate for the data in this study. However, when light scattering is introduced to simulated data, only the scatter-correction area normalization method proves robust. Using this method, common plate-based spectrophotometers found in many laboratories can be used for high-throughput screening of mAb variants and formulation stability studies.

## Highlights

• Chemical unfolding can be achieved in a high-throughput, low-cost manner.
• Equilibration time is an important parameter when measuring chemical unfolding.
• Correction for total intensity variation is critical to obtain accurate results.
• Corrections for scattered light is critical when significant amounts are present.

## Graphical abstract

**Figure:**
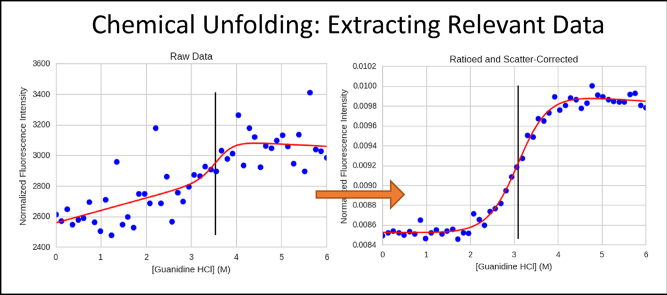

## 1 Introduction

Chemical unfolding utilizes chemical denaturants such as guanidine hydrochloride or urea to fully denature a protein or antibody. The unfolding can be monitored by several techniques including intrinsic fluorescence, circular dichroism, and UV absorption [[1], [2], [3], [4]]. By increasing the concentration of the denaturant, the measured output follows a sigmoidal relationship from a fully folded to a fully unfolded state, typically following a two-state model. From these unfolding curves, an assessment of the protein stability can be obtained by calculating the change in the Gibbs free energy of unfolding, ΔG, and the inflection point, C_1/2_, of the unfolding curve. Theoretically, the greater the ΔG and C_1/2_, the more structurally or conformationally stable the molecule [2,3,[5], [6], [7]].

Using the ΔG and C_1/2_ as a grading metric, we are screening antibody variants that have various sequence mutations with the intention of selecting a more stable molecule. By using a high-throughput version of this assay, we could potentially differentiate among hundreds to thousands of variants, identifying those variants that are more stable and those variants that are less stable than the parental antibody in a cost, material, and time efficient manner.

The ΔG can be calculated by several different methods including, but not limited to, Tanford's Model, a denaturant binding model, and the linear extrapolation method [3,8,9]. Of these methods, the linear extrapolation method is the simplest and commonly used [1,3,[10], [11], [12], [13]], and will be used as the basis for this work. A more detailed account of this method is described by Scholtz et al. and is described briefly here. The two-state, linear extrapolation method has three important assumptions: a two-state mechanism of unfolding, the unfolding event is at equilibrium, and the unfolding event is reversible. With these assumptions, the equilibrium constant can be calculated at different denaturant concentrations. Scholtz et al. describes how these equilibrium constants can then be used to directly calculate the ΔG. Scholtz et al. further describes how this calculation can be condensed into a single equation versus multiple steps. Other versions of this method are available that are applicable to three and greater state mechanisms [2,3,14].

The assumption that the system is at equilibrium is critical and indicates the need to evaluate the time necessary for equilibration. The equilibration time is determined by monitoring the measured physical parameter like intrinsic fluorescence over time and can take from minutes to hours or days to achieve [1,3,15,16]. For the purposes of high-throughput screening, it is useful to minimize equilibration time before measuring the spectra. However, we will show how equilibration time can drastically impact the ΔG and is important to both consider and report.

Measuring the intrinsic fluorescence intensity is a common technique for monitoring chemical unfolding over a range of denaturant concentrations [2,5,11,17,18]. However, there are several versions of how the fluorescence intensity output can be used when calculating the ΔG from the linear extrapolation method. These outputs include choosing a single wavelength that provides the greatest difference between the fully folded and fully unfolded fluorescence intensities, choosing the wavelength at the peak fluorescence intensity, calculating a ratio of the peak fluorescence intensity to another selected intensity, calculating a ratio of peak intensity of the protein in guanidine and PBS, and others [2,3,10,11,15,19]. The ratio method is appealing as it can correct for total intensity fluctuations such as those from variations in excitation source intensity, pathlength, concentration, and optical collection efficiency that can greatly impact a single wavelength output. However, its use is complicated by the presence of other optical effects frequently present in fluorescence spectra, such as light scattering. It can be particularly problematic to use the ratios that incorporate shorter wavelengths near the excitation wavelength, where light scattering is most intense.

A computational approach introduced in this work for the removal of light scattering is based on automated interference subtraction methods often used in the spectral domain [20,21]. These methods adjust the amount of an interfering profile until the first- or second-derivative of the spectral profile is at a minimum (i.e. is most smooth.) However, because the fluorescence and light scattering signals observed in the chemical unfolding assay are both broad in the spectral domain, such methods are not easily amenable to this assay in their original form. Instead, this work makes use of the same automated subtraction approach but applies it to the intensity profiles observed as a function of the denaturant concentration. To achieve the automated subtraction, we will describe the ratioed intensity as a function of three measured intensities: the protein signal, a reference signal, and a surrogate for the scattering signal.

Although light scattering and total intensity interferences can also be reduced by using carefully selected or engineered materials and methods [22], we are interested in developing lower-cost methods more compatible with developing an inexpensive, high-throughput chemical unfolding assay. Therefore, we investigated four different methods for analyzing the fluorescence intensity output (a single wavelength, a ratio of two wavelengths, a ratio of a single wavelength to an area, and a scatter correction plus a ratio of a single wavelength to an area) using a set of variants with different properties and calculated the ΔG and C_1/2_. In addition to defining the method(s) with minimal variation between replicates, we also evaluated simulated data with introduced light scattering. As stated previously, equilibrium of the system is central to determining the true ΔG, such that equilibration time was also investigated and shown to impact the final result. The data presented below allowed for identification of a robust method that enables a high-throughput analysis of chemical unfolding regardless of total intensity fluctuation and light scattering effects.

## 2 Materials and methods

### 2.1 Materials

Guanidine hydrochloride (Sigma, ≥99%), sodium phosphate monobasic monohydrate (JT Baker, USP grad), sodium phosphate dibasic, heptahydrate (VWR, ACS grade), sodium chloride (VWR, USP grade), LightCycler 480 Sealing Foils (Roche)and 96-well, polystyrene plates (Fisher) were used as received. Antibodies were used as received, at 1 mg/mL in PBS (20 mM phosphate, 150 mM NaC). The antibodies were produced by transient expression in an HEK293 host cell and 1-step purified via protein A affinity chromatography. Neutralized elution buffer was exchanged with PBS while normalizing the concentration to 1 mg/mL.

### 2.2 Chemical unfolding

Solutions for chemical unfolding were prepared using a liquid handling robot (Tecan Freedom EVO). Briefly, 31 different concentrations of guanidine hydrochloride in PBS (100 mM sodium phosphate, 150 mM sodium chloride) were prepared by the robot from a 7 M guanidine hydrochloride stock solution in PBS. The final concentrations used were: a range of 5.5–4 M in 0.5 increments, 3.8 M, a range of 3.6–1.7 M in 0.1 M increments, a range of 1.5–0.5 M in 0.25 M increments, and 0 M. Then, 190 μL of each of the guanidine stock solutions were transferred to a well of a 96 well plate. 10 μL of a 1 mg/mL antibody stock solution was added to each well and mixed by pipetting five times. The plate was then sealed using sealing foils to prevent evaporation and centrifuged at 3000 rpm for 2 min at 25 °C to remove any bubbles. After covering to prevent light exposure, the plate was equilibrated for a specified time at room temperature before being measured on a SpectraMax M5 plate reader. A top read, full fluorescence spectrum was collected with an excitation of 280 nm and emission from 300 to 450 nm in 1 nm steps.

### 2.3 Spectral integrations, ratios, and automated removal of light scattering

Initially, a qualitative assessment of the amount of scatter present in each sample, the scattering ratio, was performed by integrating the intensity from 300 to 305 nm and dividing it by the intensity integrated from 305 to 320 nm. In the absence of scatter, this scattering ratio will remain constant even across different protein signals but will change with changes in the relative amounts of scatter and protein change. Thus, the ratio is an effective approximation of the amount of scatter present in each spectrum.

For the quantitative assessment of protein unfolding, the intensity at 372 nm was selected as the “signal intensity”. This wavelength exhibits large differences in intensity between the folded and unfolded protein states. Simultaneously, the intensity from 320 to 440 nm was integrated and considered the “reference intensity” and the intensity from 300 to 320 nm was integrated and used as the “scatter intensity” in the spectrum.

Taking these three intensities (signal *I*_*s*_, reference *I*_*r*_, and scatter *I*_*c*_), the intensity corrected for both total intensity fluctuations and the contribution of scatter in the signal and reference wavelengths, *I*_*corrected*_, can be calculated using Equation 1

(1) $$I_{corrected}=\left(I_{s}-{\text{α}}_{1}I_{c}\right)/\left(I_{r}-{\text{α}}_{2}I_{c}\right)$$

where the *α*_*1*_ and *α*_*2*_ values are scalar weights which define the ratio of scatter observed at the signal and reference regions of the spectrum, respectively. The values for these weights must be determined for a given denaturization run by minimizing the sum-of-squares (ssq) of the first derivative of *I*_*corrected*_ versus denaturant concentration, as shown in Equation (2), where n is the total number of samples in the denaturant curve and *I*_*corrected,i*_ is the corrected intensity observed for the *i*^*th*^ sample in the denaturant curve.

(2) $$ssq=\sum_{i=1}^{n-1}{\left(I_{corrected,i}-I_{corrected,i+1}\right)}^{2}$$

The wavelengths representing the scatter intensity were selected because it is the region of the spectrum where the contribution of scatter is the largest relative to the protein. After scaling, it can be used as an approximation of the amount of scatter present in other regions of the spectrum.

### 2.4 Calculating ΔG and C_1/2_

The ΔG was calculated based on the equation described by Scholtz et al. [1] and shown below as Equation (3). All terms in this equation as are defined in the original work. From this equation, the C_1/2_ was also determined by calculating the fit to the corrected intensity then calculating the crossing point of the second derivative of those fit values.

(3) $${\hat{I}}_{corrected}=\frac{\left(I_{f}^{o}+m_{f}\left[D\right]\right)+\left(I_{u}^{o}+m_{u}\left[D\right]\right)\exp\left(\left(m\left[D\right]-\text{Δ}G\right)/RT\right)}{\left(1+\exp\left(\left(m\left[D\right]-\text{Δ}G\right)/RT\right)\right)}$$

### 2.5 Creation of simulated data

A series of simulated denaturing datasets were created to help verify the utility and unbiased nature of the correction method. First, three spectra were isolated from experimentally-measured chemical unfolding data:

(1) a spectrum of the mAb in its folded state at low denaturant concentration with empirically low scatter contribution (scatter level determined visually);
(2) a spectrum of the same mAb in its unfolded state at high denaturant concentration with empirically low scatter contribution; and
(3) a spectrum of pure scatter contribution isolated by subtracting two similarly-composed mAb solutions with empirically-different levels of scatter.

Using experimentally-realistic estimates for the ΔG (Δ*G* = 5.435 kcal/mol) and slope (*m* = 1.769 counts L/mol) and Equation (3), an ideal unfolding curve was created. To this, a small amount (1%) of normally distributed noise was added to represent experimental error in protein handling and denaturant concentration. Using the end points as fully folded and fully unfolded species and the isolated folded and unfolded spectra, a series of composite spectra were created for each denaturant concentration.

To these spectra, random amounts of the scatter spectrum were added using a pareto distribution with a shape (tail index) of 1 and a scaling factor of 10% of the scatter spectrum. Finally, total intensity fluctuations were introduced by scaling each spectrum in the denaturant profile by a random factor using a normal distribution with a standard deviation of 2% of the total intensity. The scatter and total intensity variation profiles were regenerated 50 times to simulate 50 repeat experiments on the same mAb denaturant profile.

## 3 Results and discussion

**Fig. 1:**
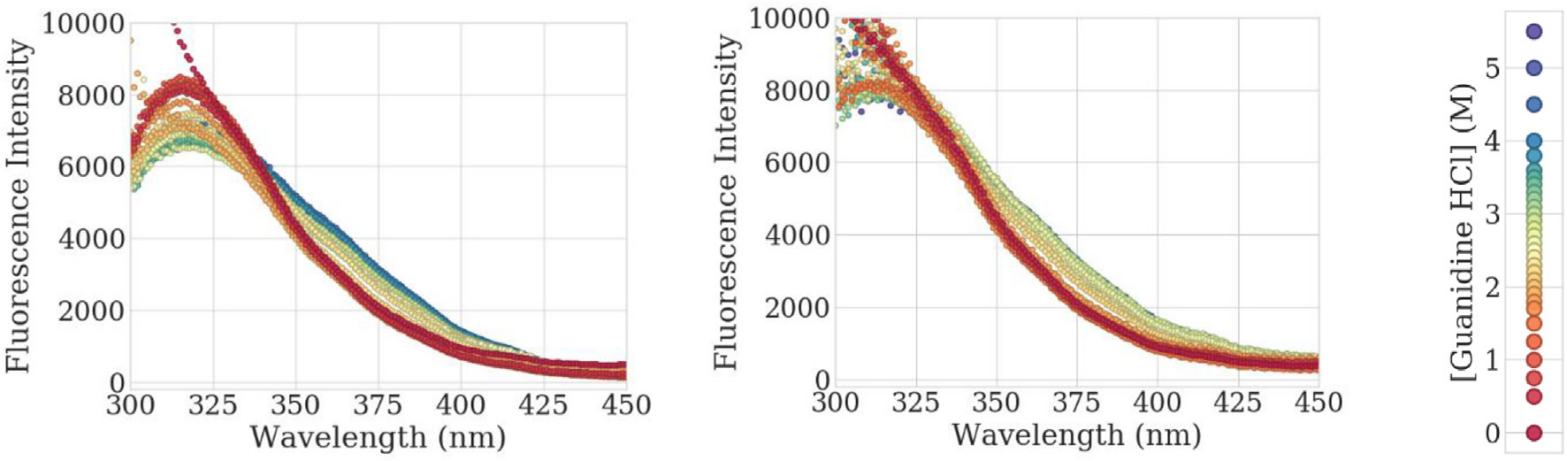
Full intrinsic fluorescence spectrum of mAb1 completed in duplicate across multiple denaturant concentrations at 24 h. Denaturant concentration is given in molarity in the legend.

There are multiple methods reported for using the intrinsic fluorescence intensity output to create chemical unfolding curves and to calculate ΔG. One example is to use the peak fluorescence intensity that occurs around 320 nm. However, if scattering is present in the spectrum, as observed in the short wavelengths in Fig. 1 for mAb1, the chemical unfolding curves are detrimentally impacted, which then leads to an imprecise ΔG value.

**Fig. 2:**
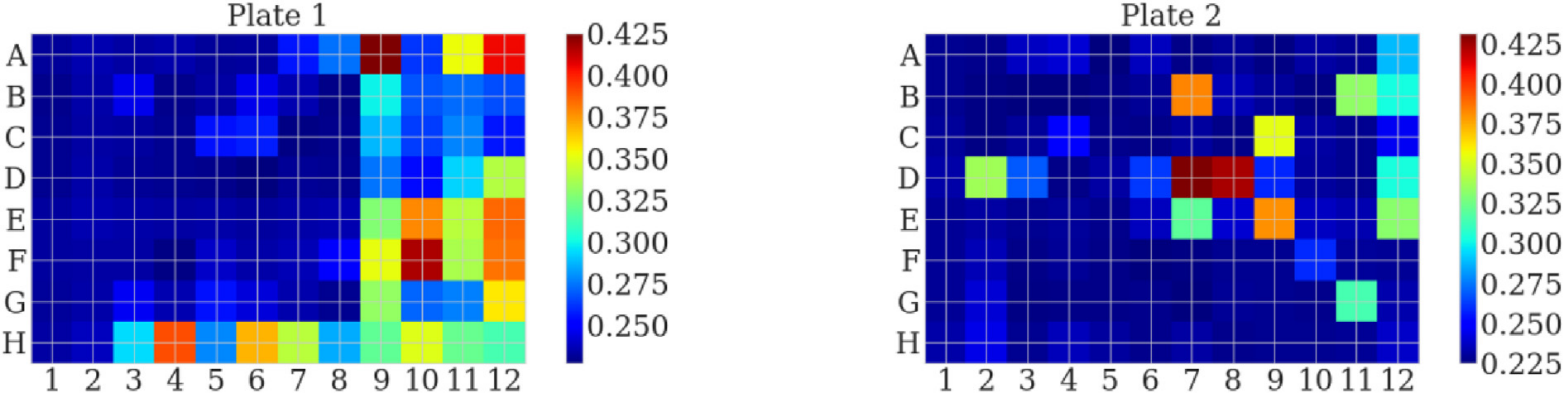
A visualization of the scattering ratio across each well across a plate completed in duplicate. The color scale shows the magnitude of scattering calculated as described in the methods where dark red is the largest scatter observed and dark blue is the lowest scatter observed. (For interpretation of the references to color in this figure legend, the reader is referred to the Web version of this article.)

**Fig. 3:**
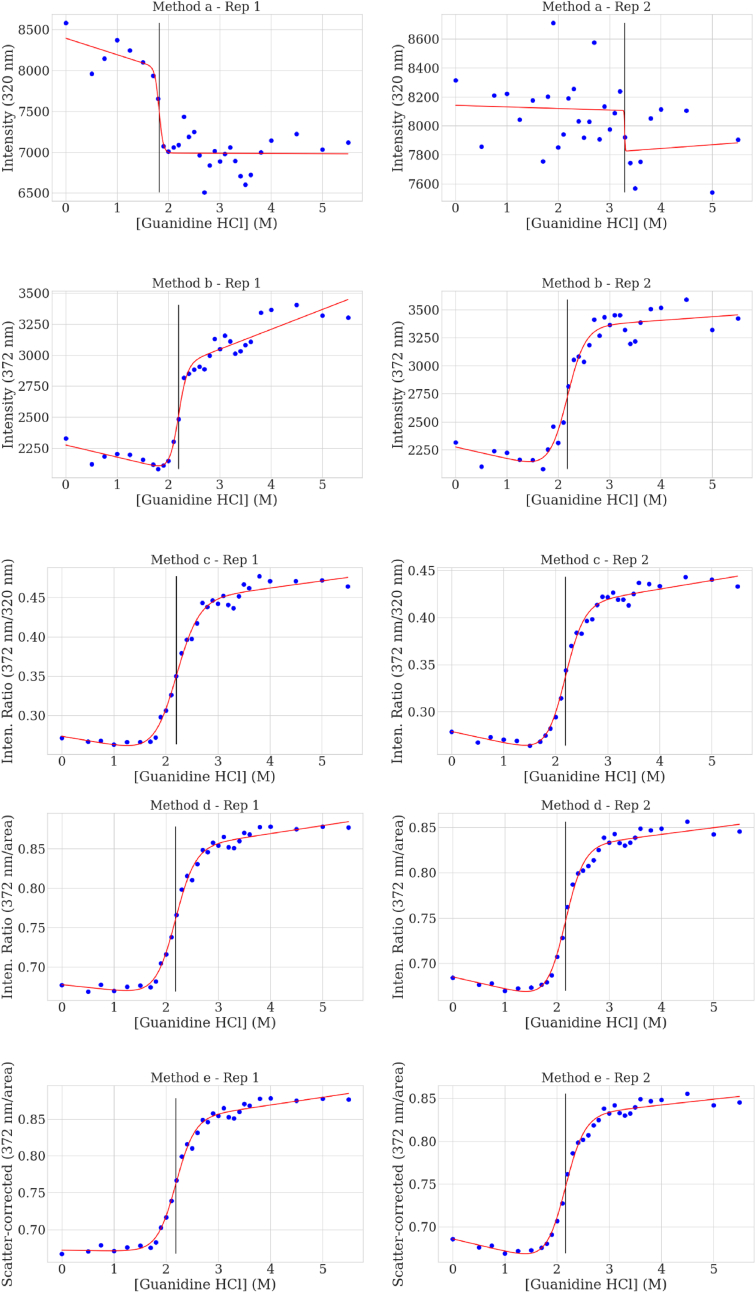
The chemical unfolding curves calculated for mAb1 at 24 h equilibration based on the intrinsic fluorescence a) at 320 nm, the approximate spectrum peak maximum, b) at 372 nm, c) using the ratio at 372 nm/320 nm, d) using 372 nm to the area normalization, and e) using the scatter correction-area normalization at 372 nm. Raw data are represented by dots, the calculated curves are from Equation (3), and the vertical lines indicate the calculated C_1/2_.

Fig. 1 shows that even between duplicates of a single sample plated by automation, the scattering can be quite variable. This variation is well represented in Fig. 2, which depicts the scattering ratio as a function of well placement for two plates with the same antibody. The scattering ratio is random in the plates without any noticeable pattern relative to well placement. While the liquid handling robot helped to reduce variability introduced by plating by hand (data not shown), there is still some inherent, random variation in each sample replicate. When the ΔG is calculated for mAb1 using the intrinsic fluorescence at 320 nm, none of the three replicates fit a standard sigmoidal curve such that the fit parameters can be interpreted in a physical sense, as shown in Fig. 3a. This indicates that, without some correction, the raw intensity values cannot be used to accurately determine ΔG.

Another method for calculating the ΔG is to use a single wavelength that provides the greatest difference in fluorescence intensity between the fully folded and fully unfolded states. This will move the wavelength of interest towards longer wavelengths around 370–380 nm for the antibodies studied in this work. These longer wavelengths are farther away from the scattering that is strongest at short wavelengths, so scattering is less impactful on these profiles. As shown in Fig. 3b, this wavelength produced data that follow a sigmoidal curve and a final ΔG value of 10.5 ± 4.1 kcal/mol. This result is more precise than using the 320 nm wavelength calculation. However, variability within the data is still present as observed in the standard deviation and by the deviation of the raw data points relative to the fitted chemical unfolding curve. We attribute this variability to small changes in the slope of the folded and unfolded regions of the curve that have a large impact on the ΔG calculation.

Using a ratio of two selected wavelengths such as a selected wavelength to the peak fluorescence wavelength can help correct for some variations due to total intensity fluctuations. When the ratio of 372 nm–320 nm is used (Fig. 3c), the data points conform closer to the calculated chemical unfolding curve and results in a more precise calculation for the ΔG, 6.9 ± 1.0 kcal/mol. While this is a vast improvement, this method is still reliant on a single emission wavelength near the excitation source and is influenced by the noise and scattering embedded in that signal. We propose the following to correct for these two sources of interference.

First, to address the issue of noise, a window of multiple points is integrated for the reference intensity to reduce the effect of random noise (relative to the use of a single reference wavelength.) Second, to address the issue of scatter in the signal, the corrected intensity and assessment criteria described by Equations (1), (2) can be used. These equations take advantage of the different spectral fingerprints of scattering and intrinsic fluorescence and, also, the expected relationship between spectral profiles at similar denaturant concentrations.

The rationale behind Equation (1) is that we know some portion of the sample and reference intensities are due to scatter. The exact amount of scatter signal present at any wavelength and integrated into the three measured intensity values (*I*_*s*_, *I*_*c*_, and *I*_*r*_) depends on the total number of scattering events scaled by the scattering profile versus wavelength. In Equation (1), the *α*_*1*_ parameter approximates the ratio between the scattering included in *I*_*s*_ and *I*_*c*_ such that subtracting *α*_*1*_*I*_*c*_ removes the scattering present in the *I*_*s*_ value. Similarly, *α*_*2*_ approximates the relative amounts of scattering in *I*_*r*_ and *I*_*c*_.

To find the *α* values, we assume that for a given experiment the relative scattering profile is consistent such that the same *α* values will apply to all spectra measured from a given plate and/or denaturant profile; the total amount of scatter present might vary but not the scatter profile. Thus, the value for *α*_*1*_ and for *α*_*2*_ can be found by using a standard non-linear optimization on the two values to minimize Equation (2). In practice, similar or identical *α* values would be expected for all data collected on a given instrument and experimental configuration, although we do not impose such constraints in this implementation.

The results of applying the first part of this approach, the wavelength to area normalization, are shown in Fig. 3d. In general, more points are closer to the chemical unfolding curve compared to the wavelength ratio method. The ΔG value is 7.2 ± 0.4 kcal/mol, a tighter set of triplicates than the wavelength ratio. When the complete method of scatter correction-area normalization is applied, as shown in Fig. 3e, the measured data more closely follows the sigmoidal curve. Although the differences appear minor, the impact these have on the recovered ΔG is significant, which is now calculated to be 7.4 ± 0.09 kcal/mol. With more precise values, it becomes easier to differentiate among variant candidates.

To demonstrate that this approach removes the effects of scatter and total intensity variations without biasing the recovered ΔG or C_1/2_, the simulated denaturing datasets containing introduced light scattering effects were analyzed. Analyzing the underlying simulated unfolding curve (prior to simulation of mixture spectra), we recover the “target” values shown in Table 1. Given that these results are the true results for this experiment, bias from these values represents the error of each spectral correction method.

**Table 1:** The mean and standard deviation (shown in parentheses) of the ΔG, slope (m in Equation (3)), and C_1/2_ recovered for all 50 simulated replicates.* These values exclude 2 replicates for which C_1/2_ could not be found within the concentration range.

	*Δ*G (kcal/mol)	Slope (counts L/mol)	C_1/2_ (M)
Target:	5.65	1.88	3.02
Single wavelength (372 nm):	43 (101)	14 (30)	3.07 (0.33)*
372nm/320 nm:	39 (233)	7 (39)	3.03 (0.43)
372nm/area:	5.62 (0.49)	1.87 (0.17)	3.02 (0.03)
Scatter and area ratio:	5.67 (0.19)	1.89 (0.06)	3.01 (0.01)

**Fig. 4:**
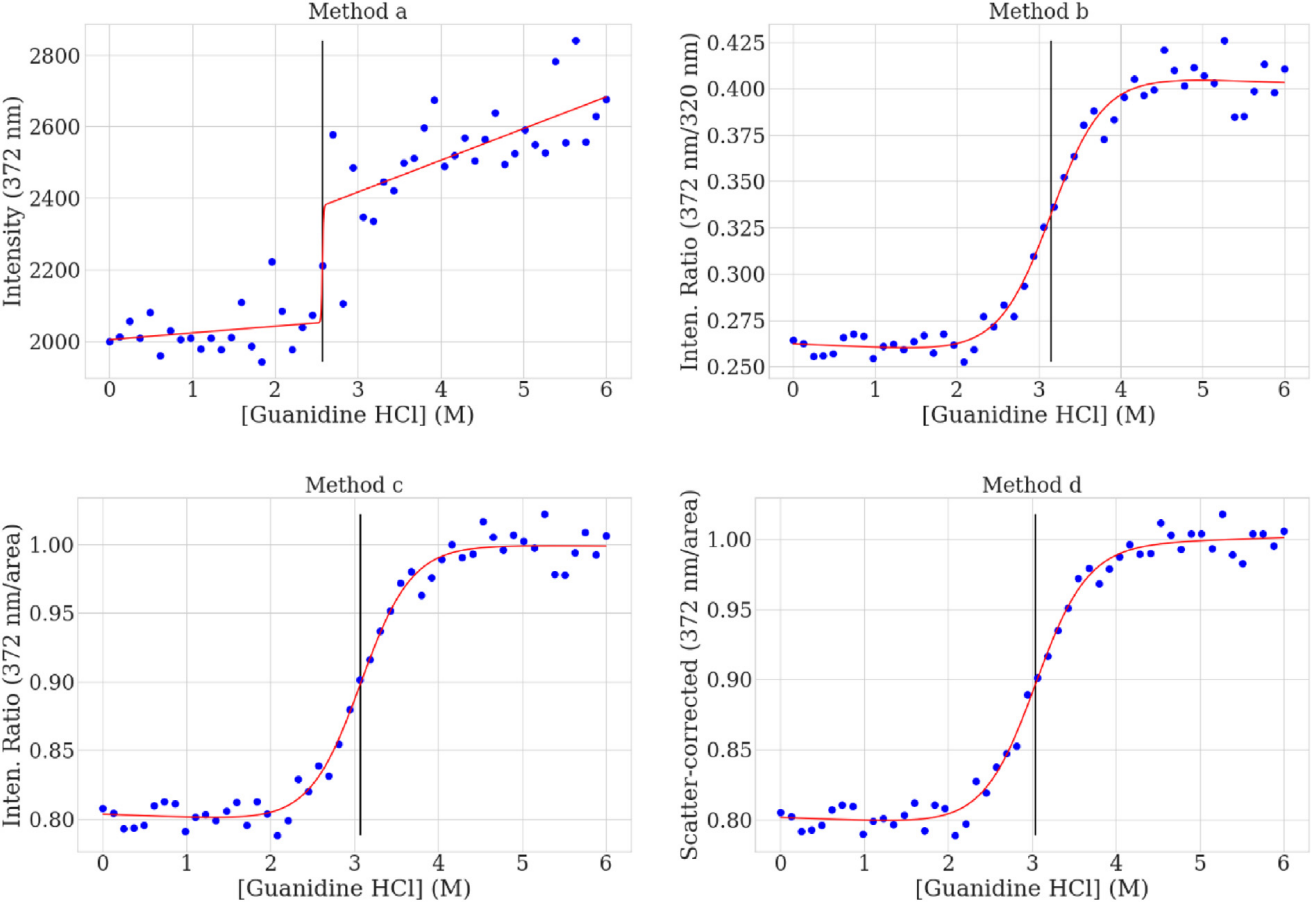
The chemical unfolding curves calculated for one replicate of the simulated data based on the simulated intrinsic fluorescence using a) 372 nm fluorescence only, b) the ratio of 372 to 320 nm, c) the ratio of 372 nm to the integration of 320–400 nm, and d) the scatter correction-area normalization at 372 nm.

An example of a denaturant profile analyzed using four different methods is shown in Fig. 4. The methods were (a) using raw intensity at the sample wavelength, (b) the ratio of the sample wavelength to a single wavelength reference, (c) the ratio of the sample wavelength to a reference area, and (d) the scatter-corrected ratio. When all 50 replicates are analyzed, and the standard deviation of the recovered parameters is calculated, as shown in Table 1, the impacts of scatter and total intensity variation can be seen. The values calculated from the single wavelength method are clearly biased and imprecise. The single-point to single-point ratio values are equivalently poor. The results calculated from using the area-ratio method are accurate on average, but their standard deviation is 2–3 times greater than those using the scatter-correction method, indicating that for normal situations when it is impractical to perform many replicates, the accuracy using the scatter correction is more likely to be better. Interestingly, the C_1/2_ is largely unaffected by the scatter-correction method, although the standard deviation does decrease slightly with the area ratio and scatter-corrected ratio. Most importantly, these results demonstrate that the scatter-correction method imparts no analysis bias.

**Fig. 5:**
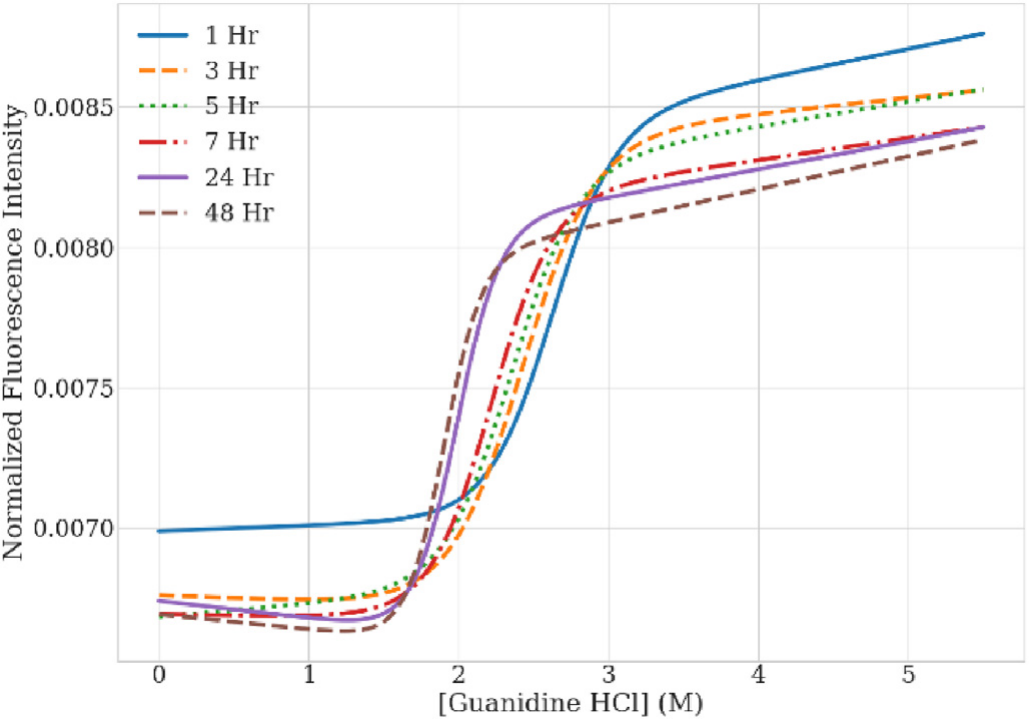
The chemical unfolding curves for mAb1 measured over 48 h.

Now that a correction method has been established which does not bias the recovered ΔG, the critical assumption of equilibrium can be examined. Fig. 5 shows a time course study of the chemical unfolding curves for mAb1 over 48 h. The 1 h curve is distinctly offset in initial normalized intensity compared to all of the other curves. As more time is allowed for equilibration, the baselines and transition region from folded to unfolded start aligning as shown for the 24 and 48 h curves. Table 2 lists the calculated ΔG and C_1/2_ values. The values fluctuate in the beginning, appear to settle at 5 and 7 h, but rise again and level out at 24 and 48 h. This drop and rise behavior may be due to multiple unfolding transitions equilibrating at different rates. Although multiple transitions were not directly observed in the unfolding curves over time, multiple transitions may be indistinguishable because of the instrument sensitivity.

**Table 2:** The ΔG and C_1/2_ values for mAb1 over time.

Time Point (hrs)	ΔG (kcal/mol)	C_1/2_ (M)
1	7.2	2.63
3	5.9	2.42
5	6.2	2.35
7	6.4	2.22
24	8.2	1.99
48	8.9	1.90

From these values, it was determined that a full 24 h equilibration should be allowed for mAb1 as an additional 24 h did not produce a large change in the recovered value. The 24 h equilibration also creates a more efficient workflow by allowing more samples to be processed per run. Multiple samples could be prepped on day one and then measured on day 2 versus only prepping a few samples, waiting 5–7 h, and reading that same day. Overall, the changes reported in Table 2 demonstrate the importance of establishing the equilibration time for each antibody to ensure the most consistent evaluation.

Using the principles we have established, we can compare the four different method outputs of calculating the ΔG using high-throughput screening. Table 3 shows a comparison of the calculated ΔG and standard deviation for 23 variants of mAb1 that contain single point mutations based on *in silico* analysis for stabilizing the molecule. As illustrated by Table 3, using a single wavelength results in a pooled standard deviation of 40.20. In contrast, the pooled standard deviation of the single wavelength ratio, the wavelength-area ratio, and the scatter correction-area normalization ratio methods are 1.10, 1.22, and 1.28, respectively. Using an F-Test, the three ratio methods are shown as equivalent, but significantly better than the single wavelength (at p<=0.05). For this specific data set, neither the concentration to concentration noise nor the wavelength-to-wavelength noise was as significant as in the simulated data, resulting in all three ratio methods being comparable for these data. Table 4 shows a comparison of the calculated C1/2 and standard deviation for the variant candidates. The relative standard deviations are notably lower compared to the relative standard deviations of ΔG. The pooled standard deviations for the C1/2 of the variants are 0.057, 0.024, 0.031, and 0.030 for the single wavelength, the single wavelength ratio, the wavelength-area ratio, and the scatter correction-area normalization ratio, respectively. The F-Test again shows that the three ratio methods are comparable (at p<=0.05) but differ from the single wavelength method.

**Table 3:** Calculated values for the ΔG at 24 h equilibration for the variants using either a single wavelength (372 nm), a ratio of two wavelengths (372 nm/320 nm), a ratio of a single wavelength to an area, and the scatter correction-area normalization method, n = 3. Also included are the pooled standard deviations for each method.

Variant	Single wavelength (372 nm)		372 nm/320 nm		372 nm/area		Scatter and area ratio	
	ΔG (kcal/mol)	Std Dev	ΔG (kcal/mol)	Std Dev	ΔG (kcal/mol)	Std Dev	ΔG (kcal/mol)	Std Dev
P	10.5	4.12	6.9	1.00	7.2	0.43	7.4	0.09
V.002	13.4	1.97	10.5	0.80	11.3	1.62	10.7	1.85
V.003	8.1	2.58	8.4	0.72	8.4	1.64	8.3	1.60
V.004	91.2	134.79	9.7	0.28	8.7	0.86	9.0	0.51
V.005	15.1	4.59	9.5	0.86	10.2	1.52	9.8	0.92
V.006	15.3	5.38	9.3	2.05	8.3	1.08	8.1	0.24
V.007	13.6	8.61	8.0	0.87	8.3	1.45	8.2	1.34
V.008	32.1	25.73	7.9	1.49	8.5	1.66	8.6	1.84
V.009	7.4	1.22	9.7	2.06	8.6	2.16	7.0	1.57
V.010	9.0	1.67	7.5	0.35	7.9	0.11	8.0	0.43
V.011	9.4	3.08	7.7	0.23	7.4	0.79	7.4	1.56
V.012	22.8	6.90	7.2	0.97	7.1	0.98	8.3	0.43
V.013	11.1	5.68	8.3	0.24	8.2	0.35	7.2	0.72
V.014	15.1	5.83	9.6	1.06	9.0	0.98	8.9	0.79
V.015	9.8	1.62	7.9	1.19	7.9	1.16	8.0	1.10
V.016	17.3	5.87	8.6	0.70	9.0	1.19	8.8	1.65
V.017	11.2	1.77	6.4	1.30	6.8	0.81	6.7	1.11
V.018	9.9	3.02	8.6	0.22	8.4	0.20	8.1	0.10
V.019	8.7	3.05	7.2	0.34	7.3	0.69	7.6	0.54
V.020	22.6	20.25	8.1	1.17	8.5	1.04	11.0	2.91
V.021	95.1	138.42	10.1	2.30	9.0	2.48	8.8	2.02
V.022	30.6	6.58	7.9	0.54	7.4	0.44	7.9	0.43
V.023	8.3	1.90	9.9	0.77	9.3	0.77	8.8	0.80
V.024	9.5	2.61	7.5	1.14	7.4	1.30	7.3	1.32
**Pooled Std Dev**	**40.21**		**1.10**		**1.22**		**1.28**

**Table 4:** Calculated values for C_1/2_ at 24 h equilibration for the variants using either a single wavelength (372 nm), a ratio of two wavelengths (372 nm/320 nm), a ratio of a single wavelength to an area, and the scatter correction-area normalization method, n = 3. Also included are the pooled standard deviations for each method.

Variant	Single wavelength (372 nm)		372 nm/320 nm		372 nm/area		Scatter and area ratio	
	C_1/2_ (M)	Std Dev	C_1/2_ (M)	Std Dev	C_1/2_ (M)	Std Dev	C_1/2_ (M)	Std Dev
P	2.186	0.010	2.197	0.012	2.166	0.013	2.151	0.005
V.002	2.219	0.095	2.204	0.007	2.183	0.025	2.177	0.028
V.003	2.086	0.007	2.127	0.004	2.094	0.009	2.044	0.023
V.004	2.183	0.090	2.225	0.054	2.197	0.067	2.169	0.033
V.005	2.182	0.058	2.200	0.014	2.182	0.020	2.157	0.023
V.006	2.117	0.033	2.075	0.011	2.048	0.039	2.030	0.020
V.007	2.303	0.057	2.275	0.040	2.243	0.042	2.218	0.034
V.008	2.176	0.109	2.187	0.041	2.150	0.049	2.124	0.018
V.009	2.403	0.064	2.450	0.008	2.403	0.025	2.311	0.078
V.010	2.185	0.042	2.174	0.004	2.137	0.014	2.084	0.023
V.011	2.186	0.016	2.183	0.020	2.146	0.023	2.110	0.033
V.012	2.159	0.022	2.208	0.012	2.179	0.018	2.140	0.018
V.013	2.165	0.088	2.176	0.007	2.146	0.009	2.078	0.031
V.014	2.372	0.016	2.339	0.033	2.315	0.049	2.285	0.022
V.015	2.216	0.050	2.210	0.008	2.184	0.020	2.164	0.026
V.016	2.108	0.014	2.153	0.018	2.119	0.011	2.084	0.012
V.017	2.104	0.095	2.038	0.037	2.016	0.040	1.983	0.016
V.018	2.188	0.007	2.195	0.002	2.162	0.010	2.142	0.035
V.019	2.119	0.040	2.084	0.047	2.048	0.056	2.024	0.036
V.020	2.142	0.038	2.214	0.020	2.181	0.024	2.149	0.017
V.021	2.168	0.056	2.222	0.005	2.190	0.013	2.145	0.006
V.022	2.126	0.033	2.194	0.009	2.177	0.025	2.154	0.031
V.023	2.218	0.055	2.226	0.008	2.198	0.015	2.167	0.044
V.024	2.175	0.063	2.159	0.025	2.130	0.023	2.133	0.008
**Pooled Std Dev**	**0.057**		**0.024**		**0.031**		**0.030**	

## 4 Conclusions

Four different methods were compared for calculating the ΔG and C_1/2_ of 23 antibody variants. It was found that the single wavelength ratio, the wavelength-area ratio, and the scatter correction-area normalization ratio were comparable for this particular data set and all were significantly better in variance and accuracy than using a single wavelength. As such, all the corrected methods enable more accurate selection of more conformationally stable molecules than would a single-wavelength method. However, if the concentration to concentration noise is significant, the scatter correction-area normalization method would result with the least noisy and most precisely fit data, as demonstrated with the simulated data. Results using this method will be more robust to experimental variations and therefore more accurate depictions of conformational stability. It stands to reason that these same improvements would be expected when using methods for fitting three or greater unfolding states, particularly because of the greater number of parameters to be fit makes these equations more susceptible to noise.

The time allowed for equilibration was also shown to have an impact on chemical denaturation and it is an important experimental condition that needs consideration. Additionally, our results show the inflection point of chemical unfolding provides a robust measurement on chemical stability that is not as strongly impacted by the method used for determining the unfolding curve.

Taken together, these results show how the combination of robust numerical analyses and carefully controlled experimental conditions can make the chemical unfolding assay a far more useful and reliable method for high-throughput screening.
